# sgRNAcas9: A Software Package for Designing CRISPR sgRNA and Evaluating Potential Off-Target Cleavage Sites

**DOI:** 10.1371/journal.pone.0100448

**Published:** 2014-06-23

**Authors:** Shengsong Xie, Bin Shen, Chaobao Zhang, Xingxu Huang, Yonglian Zhang

**Affiliations:** 1 Institute of Biochemistry and Cell Biology, Shanghai Institutes for Biological Sciences, Chinese Academy of Sciences, Shanghai, China; 2 MOE Key Laboratory of Model Animal for Disease Study, Model Animal Research Center of Nanjing University, Nanjing, China; 3 Shanghai Institute of Planned Parenthood Research, Shanghai, China; University of Minnesota, United States of America

## Abstract

Although the CRISPR/Cas9/sgRNA system efficiently cleaves intracellular DNA at desired target sites, major concerns remain on potential “off-target” cleavage that may occur throughout the whole genome. In order to improve CRISPR-Cas9 specificity for targeted genome editing and transcriptional control, we describe a bioinformatics tool “sgRNAcas9”, which is a software package developed for fast design of CRISPR sgRNA with minimized off-target effects. This package consists of programs to perform a search for CRISPR target sites (protospacers) with user-defined parameters, predict genome-wide Cas9 potential off-target cleavage sites (POT), classify the POT into three categories, batch-design oligonucleotides for constructing 20-nt (nucleotides) or truncated sgRNA expression vectors, extract desired length nucleotide sequences flanking the on- or off-target cleavage sites for designing PCR primer pairs to validate the mutations by T7E1 cleavage assay. Importantly, by identifying potential off-target sites *in silico*, the sgRNAcas9 allows the selection of more specific target sites and aids the identification of *bona fide* off-target sites, significantly facilitating the design of sgRNA for genome editing applications. sgRNAcas9 software package is publicly available at BiooTools website (www.biootools.com) under the terms of the GNU General Public License.

## Introduction

Development of tools for targeted genome editing and regulation of gene expression has significantly expanded our ability to elucidate the mechanisms of interesting biological phenomena, and to engineer desirable biological systems. The clustered, regularly interspaced short palindromic repeats (CRISPR) in combination with a CRISPR-associated nuclease 9 (Cas9) were recently demonstrated to be versatile tools for genome engineering [1]–[6]. CRISPR/Cas was first discovered as a bacterial defense mechanism against foreign (viral) DNA [7], [8]. The core endonucleases Cas9 in the type II CRISPR system has been harnessed to achieve gene mutation, DNA deletion and insertion, as well as transcriptional activation and repression, with multiplex targeting ability, just by customizing 20-nt RNA components [1]. The CRISPR-Cas9 system has been successfully used in gene targeting of different species, including the monkey, human induced pluripotent stem cells, the mouse, the rat, the zebrafish and the fly [9]–[22]. An interesting report shows that the CRISPR-Cas9 system can be used successfully to correct a genetic disease in mice with cataracts [23]. The custom-designed Cas9/sgRNA is relative simple, making this system easy to manipulate.

However, the specificity of Cas9/sgRNA needs to be carefully evaluated. Earlier studies showed that some mismatches between single guide RNA (sgRNA) and target DNA are tolerated, particularly when the mismatches are far from the 3′ protospacer-adjacent motif (PAM) [24]-[27]. In transfected cell lines, protospacers adjacent to an “NAG” PAM sequence can also be cleaved [24]. These undesired off-target effects have raised significant concerns for the use of CRISPR-Cas9 as a genome editing tool in diverse applications. To minimize off-target activity, a double nicking strategy using D10A mutant Cas9 nickase (Cas9n) was established [28]. In addition, it has been reported that the specificity of the CRISPR-Cas9 nuclease can be improved by using truncated sgRNA without sacrificing on-target genome editing efficiency [29]. No matter what methods are used, designing a high degree of specificity-targeting sgRNA is one of the more important aspects of improving the gene-editing system. A number of online and stand-alone tools have been developed, but they have different limitations. For instance, online tools only evaluate sgRNA potential off-target cleavage sites for a given species' genome. Some stand-alone tools only find CRISPR sgRNA, while others just predict candidate sgRNA off-targets.

Here we describe sgRNAcas9: a software package that can be applied to search rapidly for CRISPR target sites, and analyze the potential off-target cleavage sites of CRISPR-Cas9 simultaneously. Moreover, candidate CRISPR target sequences with high specificity will be provided to design a sgRNA expression vector. It also provides flexible output and experimentally-orientated design parameters, enabling the design of CRISPR sgRNA with high specificity for any organisms in a few hours.

## Methods

### Program overview

**Figure 1:**
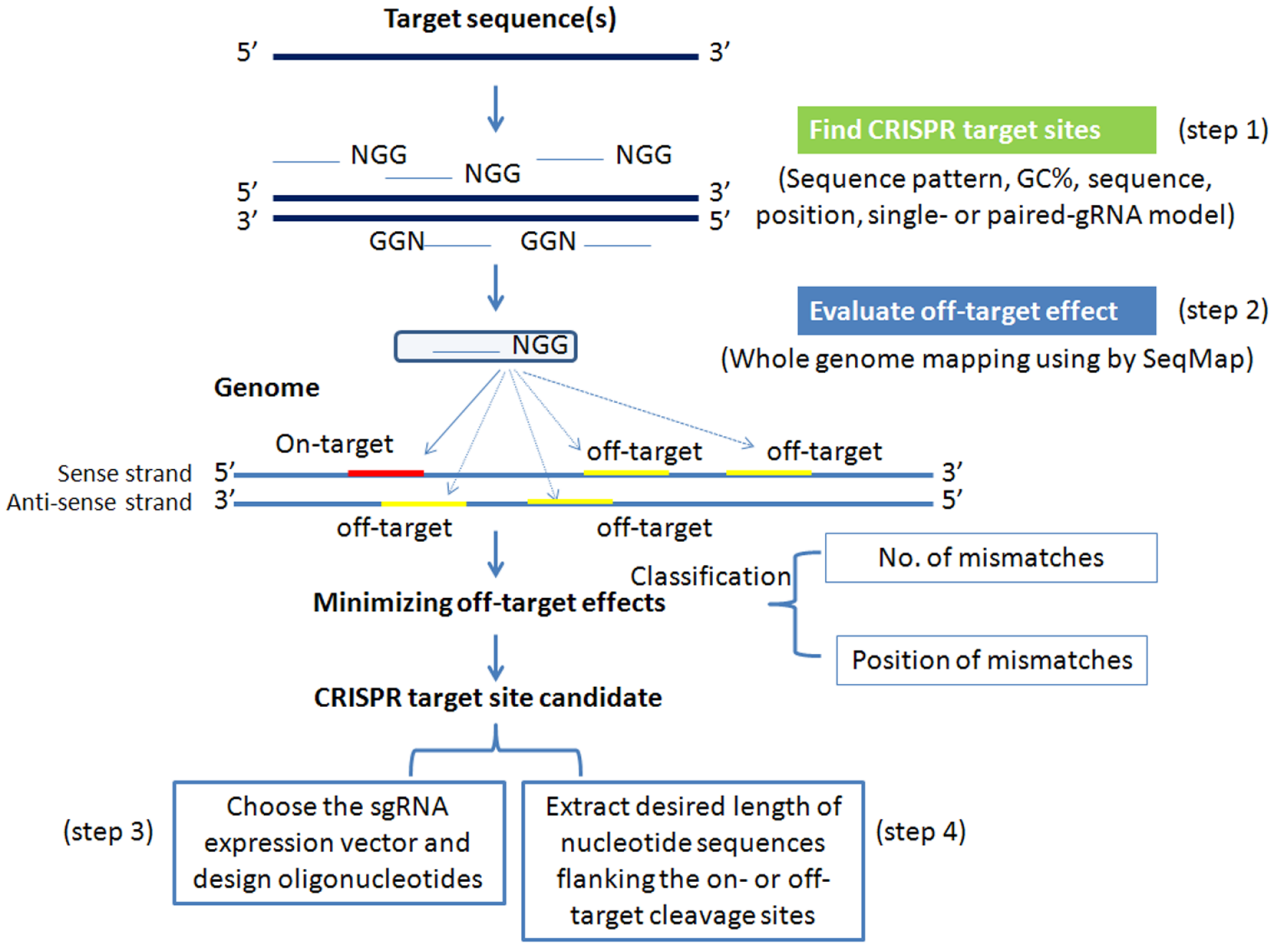
Workflow diagram of sgRNAcas9 software. Finding CRISPR target sites and off-target risk assessment includes 4 basic steps. It is noteworthy that to evaluate off-target effects exaclty, different types of potential off-target cleavage sites (POT) are classified.

sgRNAcas9 (version 2.0.6), a software package, contains seven Perl (Practical Extraction and Report Language) scripts and can run locally in Windows, Linux and Mac OS X systems (File S1). These perl scripts, each executing different tasks, are listed as follows: (1) sgRNAcas9.pl (main script), (2) format_genome.pl, (3) ot2gtf.pl, (4) pot2gtf.pl, (5) check_sgRNA_seq.pl, (6) sgRPrimer.pl, and (7) extract_targetSeq.pl. SeqMap is a tool that can map large amounts of short oligonucleotides to the genome at very high speed, making it suitable for use as an off-target predictor [30]. Herein, SeqMap, which is used as a genome-wide Cas9/sgRNA off-target searching engine, has already been included in the sgRNAcas9 software package. The main steps of the sgRNAcas9 workflow are shown in Figure 1. Step 1. Search CRISPR target sites. Step 2. Evaluate off-target effects. Step 3. Choose the sgRNA expression vector and design oligonucleotides. Step 4. Extract desired length of nucleotide sequences flanking the on- or off-target cleavage sites for designing PCR primer pairs to validate Cas9 endonucleases cleavage activity. Step 1 & 2 are performed by script format_genome.pl, sgRNAcas9.pl, ot2gtf.pl, and pot2gtf.pl; Step 3 is performed by check_sgRNA_seq.pl and sgRPrimer.pl; Step 4 is performed by extract_targetSeq.pl.

### Find CRISPR target sites

**Figure 2:**
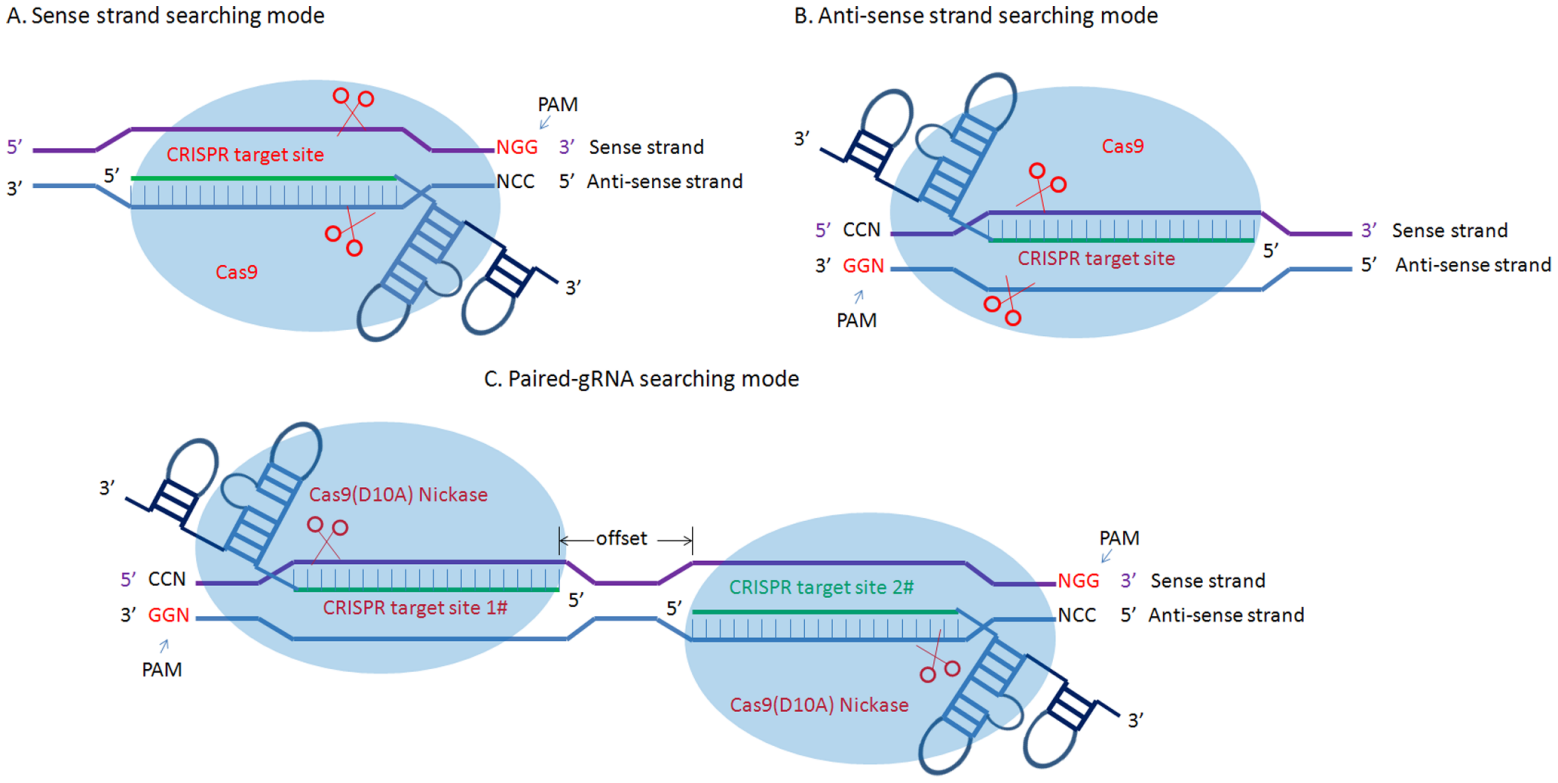
Determine the sequence of the base-pairing region on the sgRNA by the use of different searching mode. (A) Sense strand searching mode (the direction of sequence is 5′ to 3′). (B) Anti-sense strand searching mode (the direction of sequence is 3′ to 5′). (C) Paired-gRNA searching mode. Notes: different mode of sgRNA binding to target DNA strand are shown. A and B are single strand searching mode, there is also an option for using both strand searching mode (not shown).

To find CRISPR/Cas9 target sites, four searching mode (sense-strand searching, anti-sense strand searching, both strand searching, and paired-gRNA searching) are provided by using sgRNAcas9.pl program (Figure 2). No matter which mode is used, the searching pattern of CRISPR target sites is set as 5′-GGX18NGG-3′, 5′-GX19NGG-3′ or 5′-X20NGG-3′, where N and X is any base, NGG is the PAM sequence. The purpose of “G” or “GG” location at 5′ is to satisfy the requirement that sgRNA sequences should start with “G” to maintain transcript initiation, if a U6 snRNA promoter or T7 promoter is used to express a functional sgRNA. Input sequences should be provided in FASTA format with a 5' to 3' direction. Any sequence given as input file will be named as the “sense strand” in this program. Once “both strand searching mode” or “paired-gRNA searching mode” is selected, the sequence can be converted into its reverse-complement counterpart (anti-sense strand) by the sgRNAcas9 program. Genome and GTF files can be downloaded from Ensembl ftp site (http://www.ensembl.org/info/data/ftp/index.html) or NCBI website (ftp://ftp.ncbi.nlm.nih.gov/genomes/). Genome DNA sequence with FASTA headers need to be pre-treated by script format_genome.pl before running main program sgRNAcas9.pl.

Previous studies have shown that sgRNA sequences with very high or low GC content (%) are less effective against their targets [31]. To increase cleavage efficiency of a particular sgRNA, GC content should be carefully considered. To fulfill this demand, the value of GC content was set as an option argument in the sgRNAcas9.pl program. A default parameter value is provided, with a GC content range from 20% to 80% [31]. Interesting research has shown that truncated sgRNA with complementarity lengths of 17 or 18 nt can be used to improve specificity of CRISPR-Cas nuclease [29]. To meet this demand, lengths of sgRNA are set as an optional argument in the sgRNAcas9.pl program, which therefore makes it very convenient for designing truncated sgRNA. It is worth mentioning that the both strand searching and paired-gRNA searching modes are different. In order to enhance genome editing specificity, hspCas9 D10A is used in complex with paired-gRNA to generate double nicking with a 5′ overhang [3], [28]. The paired-gRNA searching mode is used to aid the user in selecting sgRNA pairs with maximized genome modification efficiency. The target loci for the sgRNA pairs must be offset with an optimal gap. sgRNA offset is defined as the distance between the PAM-distal (5′) end of the guide sequence of a given sgRNA pair. Therein, the optional argument is set to enforce the search for sgRNA targets with user-defined values. Default offsets are also provided, ranging from −2 to 32 bp (base pairs), to optimize the precision of target modification on the basis of experimental data [28]. During our manuscript's peer review, two new research reports about dimeric CRISPR RNA-guided *FokI* nucleases, which depend on the binding of two guide RNAs to DNA, have been published [32], [33]. The optional argument which is set to search for paired-gRNA is also suitable for designing two guide RNAs with the new method, when the value of the two sgRNAs distance is different. One report showed that each gRNA/FokI-dCas9 complex has a particular relative orientation with a restricted intervening spacer length of 14–17 bp [32], while another research group found that DNA cleavage by fusion of catalytically inactive Cas9 and *FokI* nuclease (fCas9) required association of two fCas9 monomers that simultaneously bind target sites ∼15 or 25 bp apart [33]. Thus, to fulfill the demand, difference parameter values need to be set carefully. The main output files which are produced by the sgRNAcas9.pl program, named “report_protospacer_single.txt” and “report_protospacer_pairs.txt”, will report all single or paired CRISPR target sequences (5′-3′). Meanwhile all the corresponding information for each target site is provided, such as start and end values, sequence pattern, GC content, sgRNA offset, etc.

### To evaluate off-target effect by classifying potential off-target cleavage sites

After CRISPR target sites have been identified, the candidate target sequences need to be evaluated for the off-target effects by alignment to the genome. This is the most critical and a time-consuming step. In this study, SeqMap was used to map full length (23 nt, including NGG PAM sequence) of CRISPR target sequences to whole genome. Several studies have demonstrated that the Cas9 tolerates mismatches between sgRNA and its target site at different positions in a sequence-dependent manner, sensitive to the number, position and distribution of mismatches [24]. Several groups have independently shown that CRISPR/Cas9 indeed induces off-target mutations, even at sites that differ by 5 nt from on-target sites in human cells [24]–[27]. In this case, the number of mismatches should be carefully determined. The optional argument of number of mismatches in the sgRNAcas9.pl program was thus set to enforce the search for sgRNA off-targets with user-defined values. The default maximum number of mismatches is set at 5 in this program.

**Figure 3:**
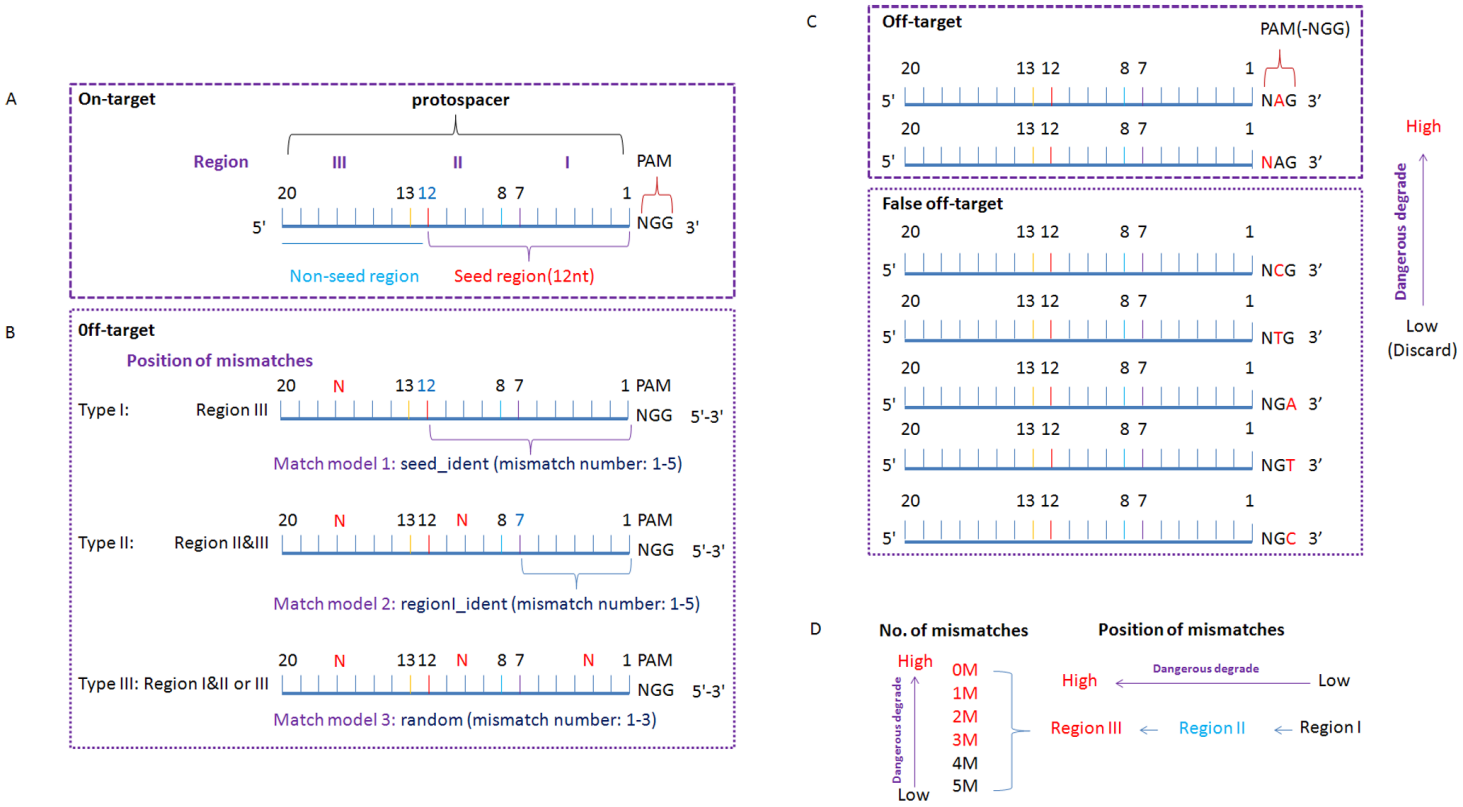
Scheme of classifying off-target cleavage sites with the sgRNAcas9 program. (A) Different segments of the target sequence. (B) The potential off-target cleavage sites (POT) were classified into three categories by count number and position of mismatches. (C) Distinction of true and false off-targets. (D) Evaluating the degree of risk of the POT. The dangerous degree of POT from low to high is dependent on the number and position of mismatches.

Previous reports have shown that Cas9 nuclease cuts 3-nt upstream of the PAM site [34]. The 12 nt upstream of the PAM site are often referred to as the seed sequence and are the most critical determinants of cleavage specificity [8], [35]. For example, a mismatch in the seed region may cause a notable reduction of the cleavage activity of Cas9/sgRNA, while mismatches in the other regions of the protospacer (the non-seed region) have a much weaker effect [5]. Another report has shown that only the first seven base pairs near the PAM site are of great importance for recognition efficiency in bacteria [36]. To describe the position and distribution of mismatches in this study exactly, target sequences were first segmented into three parts: seed, non-seed and PAM (Figure 3A). Seed and non-seed sequences were further segmented into three parts: region I (1–7 bp), region II (8–12 bp) and region III (13–20 bp). As shown in Figure 3A, the seed region contains regions I and II, while the non-seed region only contains region III.

On the basis of the above consideration, potential off-target cleavage sites can be classified into three categories from the number and position of mismatches, as shown in Figure 3B. Type I, with 1∼5 mismatched bases, are only located on region III (non-seed region); Type II, with 1∼5 mismatched bases, is located on regions II and III; Type III, with 1∼3 mismatched bases, is randomly distributed on the regions I, II and III, but with at least one mismatched base locate on the region I. The mapping result produced by SeqMap was re-analyzed and classified into three types on the above-described standards. The total numbers of mismatched bases were counted (‘N' in the PAM sequence is not counted as a mismatched base). Furthermore, as shown in Figure 3C, mismatched bases located on PAM sequences that cause a “NGG” change to “NAG”, are also not counted as mismatched bases. Furthermore, if the PAM sequence is changed to “NCG/NTG/NGA/NGT/NGC”, the corresponding predicted sequences should be discarded. In addition, the dangerous degree of potential off-target cleavage sites can be further evaluated (Figure 3D). This can be used to aid the determination of suitable CRISPR target sites.

### To select CRISPR target sites with high specificity

**Figure 4:**
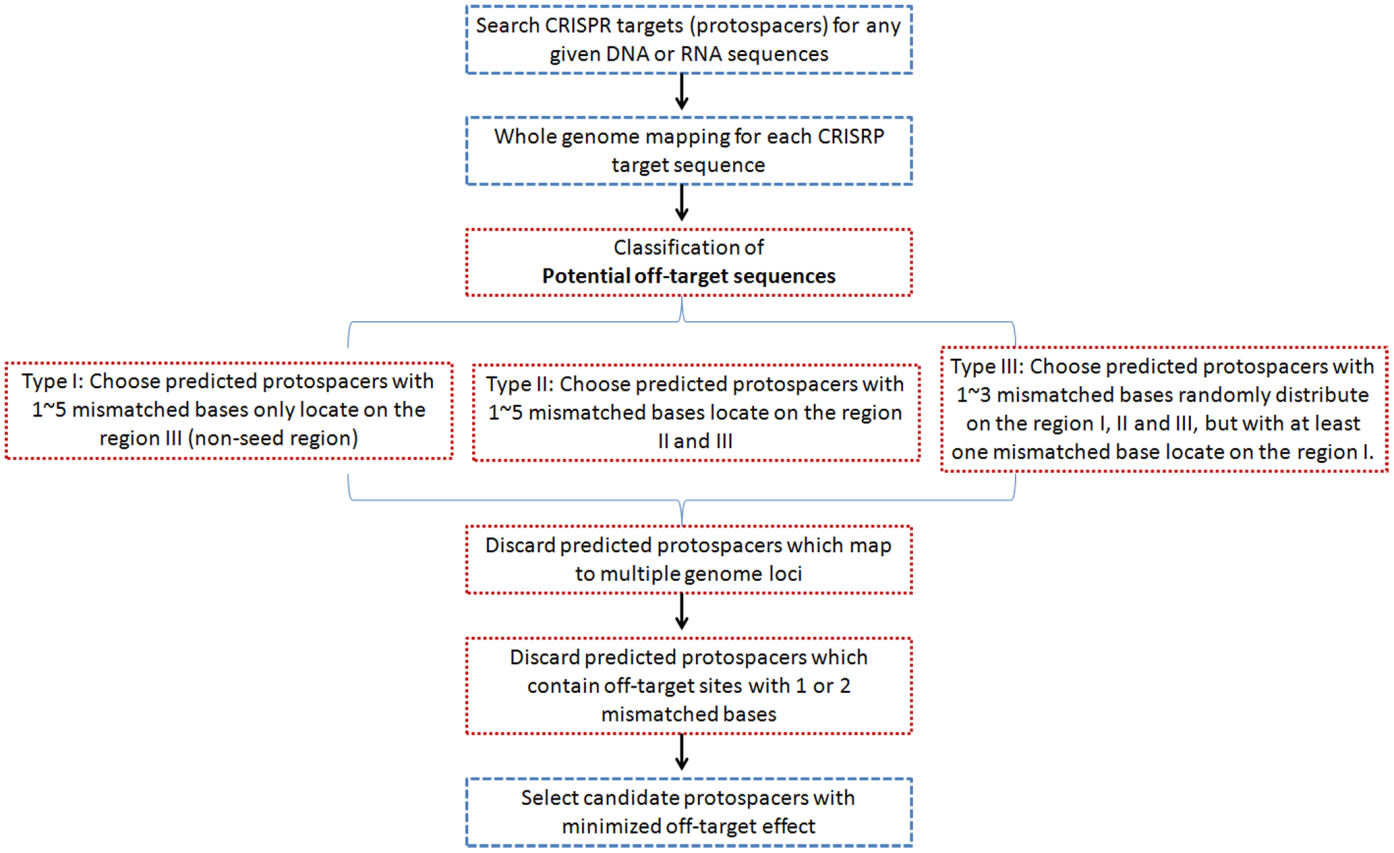
Workflow and filter criteria for selecting candidate CRISPR target sites (protospacers) with high specificity using sgRNAcas9.

After classification of potential off-target cleavage sites, candidate CRISPR target sites with minimized off-target effects can be selected. The workflow and filter criteria for selecting candidate CRISPR target sites with high specificity using sgRNAcas9 are shown in Figure 4. Predicted protospacers which are not located on the genome were first discarded. Any CRISPR target sequences which are mapped to multiple genomic loci are also discarded (Figure 4). Owing to the accurate prediction of potential off-target site, the degree of risk of the potential off-target effect was evaluated on the basis of the above-described standards (Figure 3D). For example, if sgRNAs carry single or two mismatched bases, which are especially located on the non-seed region, “off-target” cleavage may occur. Therefore, protospacers which contain off-target sites with 1 or 2 mismatched bases are discarded (Figure 4). Finally, useful information will be provided with a folder that named “Sort_POT_byID”. Each candidate sgRNA with potential off-target analysis result will be written into a separate file containing the following information – potential off-target DNA sequences with mismatched bases noted in lowercase letters, number of mismatched bases, ID, chromosome number, position, direction and type. In addition, to evaluate whether off-target sites are located in the gene coding region, perl scripts ot2gtf.pl and pot2gtf.pl can be used. Thus, the candidate CRISPR sgRNA with minimized off-target effects can not only be determined from the number of total off-target sites and potential off-target cleavage sites (POT), but also take into account the information of off-target genes. After a careful check of the specificity of sgRNA binding in the genome, CRISPR target sequences with high specificity will be selected by sgRNAcas9, and the results will be written into an output folder named “Final_report”.

### To design oligonucleotides for constructing sgRNA expression vectors and extracting target sequence from nucleotide position

To construct a sgRNA expression vector, protospacer sequences should not contain repeat sequence as follows: more than 4 continuous T nucleotides (4∼6 nucleotide poly (T) tract acts as a termination signal for RNA pol III), or other homopolymer sequences (more than 5 continuous A or C or G, more than 6 dinucleotide or trinucleotide repeats). This step can be performed by check_sgRNA_seq.pl. Once candidate CRISPR target sites are determined, selected sequences can be used to design oligonucleotides. As described above, the sequence pattern of CRISPR target sites found by sgRNAcas9.pl are 5′-GGX18NGG-3′, 5′-GX19NGG-3′ or 5′-X20NGG-3′. Therefore, the sequence of GGX18, GX19 or X20 will be extracted and used directly to design 20-nt length of sgRNAs by using sgRPrimer.pl. To describe how to use this script to batch design oligonucleotides for constructing sgRNA expression vector, the pGL3-U6-gRNA-Puromycin vector (modified from Addgene 51133) was selected as an example, which is designed for expressing customizable sgRNA under control of the U6 promoter. Annealed oligos were cloned into the vector at a *Bsa* I restriction site. To facilitate cloning of the 20 bp target sequence, extra bases need to be added to the ends. In this study, ‘accg’ was added to the 5′ end of the sense oligo and ‘aaac’ to the 5′ end of reverse complementary sequence (anti-sense oligo). Then, equal amounts of the sense and anti-sense strands were synthesized and annealed to generate the ds-oligo. This product can be easily ligated into the digested pGL3-U6-gRNA-Puromycin vector.

To investigate on- or off-target cleavage effects, certain lengths of predicted sequence need to be extracted from the genome by nucleotide positions. Then cleavage sites can be validated by using the T7 endonucleases I (T7E1) assay or sequencing. This is another time-consuming step. To raise experiment efficiency and save time, extraction of target sequence by nucleotide position can be performed by extract_targetSeq.pl. The length of sequences extracted from genome was set as an optional argument in this program. A default parameter value was provided to extract DNA fragments up to 1,000 bp in length. Then the sequence was used as a template to design PCR primer pairs for validation of the Cas9 cleavage effect.

## Results and Discussion

### Software performance testing

After description of the technical details of how sgRNAcas9.pl (main script) is implemented, here are some examples demonstrating its usage and capabilities. Computer performance is as follows: Dual Core Processor (Intel(R) Core(TM)i3-2130CPU@3.40GHz 3.40GHz), 8 GB RAM memory, System platform: ubuntu 12.04 LTS (64-bit). SeqMap version: 1.0.12 64-bit (x86_64, Linux). The human *Emx1* gene (NCBI accession number: NM_004097.2) was selected as a simulation example (Text S1). Herein, full length CDS of *Emx1* gene is divided into three exons, their length being 898 bp, 185 bp and 1105 bp. Human genome DNA sequence (Genome assembly: GRCh37, GCA_000001405.14) was downloaded from Ensembl ftp website and the size of the whole DNA genome is 3.1 GB (ftp://ftp.ensembl.org/pub/release-74/fasta/homo_sapiens/dna/). To find CRISPR/Cas9 target sites for *Emx1* gene, four searching mode were tested. From a Windows or Linux command-line interface, the sgRNAcas9 program can be run easily. For example, if the user plans to design paired gRNA to target *Emx1* gene, paired-gRNA searching mode is recommended. The command-line is “perl sgRNAcas9.pl -i hEMX1_example.txt -x 20 -l 40 -m 80 -g genome_example.fa -o b -t p -v l -n 5 -s 5 -e 35”. The user can also use simple command with default options (length of sgRNA: 20 nt, GC content: 20% to 80%, number of mismatched bases: 5, sgRNA offset: −2 to 32 bp): “perl sgRNAcas9.pl -i hEMX1_example.txt -g genome_example.fa -o b -t p -v l”. For detailed information about running the sgRNAcas9 program, please see README file or BiooTools website (www.biootools.com). Comparison of results produced by different searching mode showed a variation in the number of candidate CRISPR target sites provided (Table 1). Notably, the running time of different searching mode was not increased as the number of CRISRP target sites increased (Table 1). Undoubtedly, the speed will be increased if high performance computers are used.

**Table 1:** Running time for finding CRISPR target sites and searching off-targets.

	*Exon 1*	*Exon 2*	*Exon 3*	*Total No. of sgRNA*	*Time*
**Sense strand searching mode**	54	11	138	203	*8522* *s*
**Anti-sense strand searching mode**	73	17	92	182	*8314* *s*
**Both strand searching mode**	127	28	230	385	*9496* *s*
**Paired-gRNA searching mode**	148 pairs	37 pairs	11 pairs	197 pairs	*7621* *s*

Note: Default parameter is used to perform different searching mode by sgRNAcas9.pl. Length of exon: exon 1 (898 bp), exon 2 (185 bp), and exon 3 (1105 bp).

### Example of output

**Figure 5:**
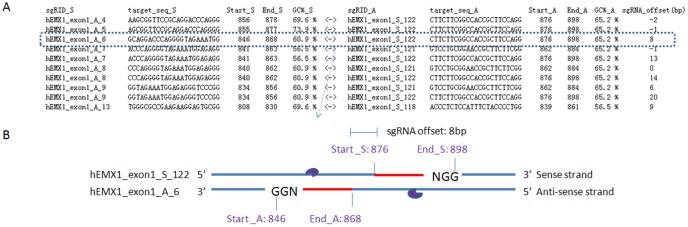
Example of CRISPR on-target sequences found by sgRNAcas9. (A) The paired-gRNA target sites of *Emx1* gene found by sgRNAcas9 program and the paired-gRNA searching mode. (B) Illustration of one pair sgRNA targeting at exon1 of *Emx1*.

**Figure 6:**
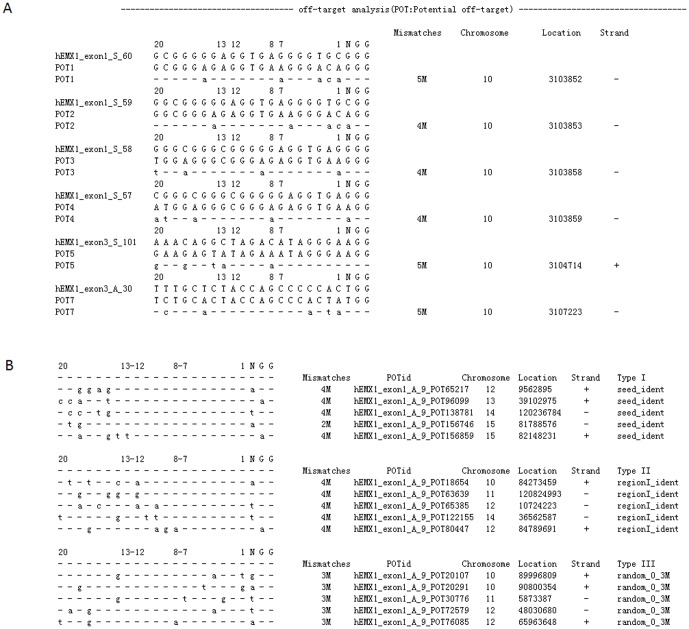
Example of the classification of potential off-target sequences by sgRNAcas9. (A) Searching off-target sites for each sgRNA targeting at human *Emx1* (hEMX1) gene. (B) Classifying POT by number and position of mismatches into three types. Notes: “seed_ident”, strand for seed region, was to identity to on-target sites (Type I). “region I_ident”, strand for region I identical to on-target site (Type II). “random_0_3M”, strand for regions with 1∼3 mismatched bases randomly distributed on the region I, II and III, but with at least one mismatched base located on the region I (Type III).

Ten text files and eight folders are produced after running the sgRNAcas9.pl program. For detailed illustration of example output, enter our BiooTools website. Here, we describe in detail how to organize the result. As described above, human *Emx1* gene was selected as an example to test the program performance. Different results of CRISPR target sites produced using sgRNAcas9 in different searching mode are listed in Tables S1, S2, S3 and S4. As shown in these Supplemental Tables, information of the ID number of the target site, start and end values, sequences, patterns and GC content is provided. A typical example of results from the paired-gRNA searching mode is shown in Figure 5A, the value of sgRNA offset being given. Illustration of one pair sgRNA targeting at exon1 of *Emx1* is shown in Figure 5B. Next, each sequence in the Table was aligned to the whole genome to perform a specificity check. Mapping the result produced by SeqMap (file name “seqmap_output.txt”) was re-analyzed, and the result was written into a file “search_OT.txt”. An example illustration of re-analyzed mapping output is shown in Figure 6A. The information of on- and off-target sequences, the number of mismatched bases, chromosome number, location and strand is given. The total number of off-target sites for each CRISPR target sequence is given (Table S5). The number of total off-target sites ranged from 53 to 19366.

Subsequently, potential off-target cleavage sequences were classified. The result is written into the files and distributed to different folders, such as “Type_I_POT”, “Type_II_POT”, and “Type_III_POT” by the sgRNAcas9.pl program. An example result is shown in Figure 6B. Three types of potential off-target sites with all relative information can clearly be seen. In addition, the total number of the potential off-target sites for each CRISPR target sequences are given (Table S6). The number of potential off-target sites ranged from 1 to 2285. Interesting, by comparing the total number of off-target sites with potential off-target sites (POT) for each CRISPR target sequence (Table S7), we found that some target sequences with a large total number of off-target sites contain a relatively small number of POT after classification. For example, the total number of off-target sites of hEMX1_exon2_A_12 was 152, while its potential off-target sites were only 1. To exclude CRISPR target sequences containing off-target sites with 1 or 2 mismatched bases, potential off-target sites were reanalyzed (Table S8). As listed in Table S8, protospacers containing potential off-target sites with 1 or 2 mismatched bases, or having more than one perfect match target site, were extracted. Subsequently, the remaining CRISPR target sites were selected and are listed in Table S9. Finally, three optimized candidate CRISPR target sequences were chosen for each exon of the *Emx1* gene with a relatively lower total number of off-target sites and potential off-target sites for designing the sgRNA expression vector (Table S10).

### Comparison with other CRISPR sgRNA design tools

A few online or stand-alone tools have been developed to design CRISPR target sites or predict off-target sites. Online tools “Cas9 Design” (http://cas9.cbi.pku.edu.cn/index.jsp) [37] and “CRISPR/Cas9 gRNA finder” (http://spot.colorado.edu/~slin/cas9.html) can be used to design single or paired sgRNAs, but does not find off-targets. Cas-OFFinder (http://www.rgenome.net/cas-offinder/portable) is a web and stand-alone tool, which very rapidly finds off-targets for individual CRISPR sgRNA, but does not find candidate sgRNAs [38]. Another stand-alone tool is CasOT, which can be used to find candidate sites from input sequence as well as finding or printing out potential off-target sites, and it attempts to 'score' the effect of the off-target by notifying if it is placed inside a coding exon [39]. Other online tools, such as ZiFiT (http://zifit.partners.org/ZiFiT/ChoiceMenu.aspx) [24], “Optimized CRISPR Design” (http://crispr.mit.edu/) and E-CRISP (http://www.e-crisp.org/E-CRISP/) [40] can identify all off-target sequences (preceding either NAG or NGG PAMs) across the genome. These tools can automatically rank each possible sgRNA according to its total predicted off-target cleavage; the top-ranked sgRNAs may represent those that are likely to have the greatest on-target and the least off-target cleavage. Although these online tools are powerful, have user-friendly interfaces and are easy to use, only a few species' genomes are provided, which limits their application. For instance, web tools ZiFiT, “Optimized CRISPR Design” and E-CRISP only provide 5, 15, 18 species' genomes, respectively. Detailed information about comparison of different CRISRPCas9 design tools is listed in Table S11.

In this study, a novel open-source application named sgRNAcas9 is described, which contains seven Perl scripts that can be reliably used to design scored sgRNA expression vectors. One script formats genomic sequence FASTA files (format_genome.pl) in that it only removes everything but the chromosome name and the sequence information, the second (main) script sgRNAcas9.pl, extracts CRISPR target sequences and – with the help of an external software package – evaluates off-target effects. Two scripts ot2gtf.pl and pot2gtf.pl can be used to check off-target sites, and whether they are located in the gene-coding region, and the remaining three scripts check_sgRNA_seq.pl, sgRPrimer.pl and extract_targetSeq.pl are involved in PCR-primer pair design for cloning the sgRNA into specified expression vectors and primers that bind to genomic regions around the on-target sites; this is useful to evaluate the CRISPR/Cas9 activity after the experiment has been performed. In comparison with online-tools such as “Optimized CRISPR Design” (http://crispr.mit.edu/), the advantage of the current package is: (a) local execution (data privacy); (b) flexibility of parameter settings; (c) wide choice of any species' genome; (d) the workflow comprises all major computational steps required for CRISPR/Cas9. In addition, compared with stand-alone tools such as CasOT, besides being used to find CRISPR sgRNA, and predict off-targets simultaneously, sgRNAcas9 has a number of extra features. For instance, candidate CRISPR sgRNAs with minimized off-target effects can be determined by means of balancing the total off-target sites and potential off-target cleavage sites by using sgRNAcas9. To save time and improve efficiency, the users can perform a batch design of oligonucleotides for constructing sgRNA expression vectors, and extract the desired length of nucleotide sequences flanking the on- or off-target cleavage sites, making it convenient for constructing sgRNA expression libraries. The latest available sgRNAcas9 can also be used to check whether an off-target is inside the coding sequence. However, in comparison with web tools, users with non-bioinformatic background may face hurdles in running this program. To solve this problem, a step-by-step guide to facilitate the use of sgRNAcas9 is provided, which can be downloaded from our website, and a user-friendly interface version of sgRNAcas9 will be developed in the future.

## Supporting Information

Table S1 CRISPR target sites of human *Emx1* gene found by sgRNAcas9.pl and use of the anti-sense strand searching mode.
(XLS)

Table S2 CRISPR target sites of human *Emx1* gene found by sgRNAcas9.pl and use of the sense strand searching mode.
(XLS)

Table S3 CRISPR target sites of human *Emx1* gene found by sgRNAcas9.pl and the use of both strand searching mode.
(XLS)

Table S4 CRISPR target sites of human *Emx1* gene found by sgRNAcas9.pl and use of the paired-gRNA searching mode.
(XLS)

Table S5 Total number of off-target sites (OT).
(XLS)

Table S6 Total number of potential off-target sites (POT).
(XLS)

Table S7 The comparison of the total number of OT and POT for each CRISPR target sequence.
(XLS)

Table S8 To find POTs containing 1 or 2 mismatches.
(XLS)

Table S9 To select candidate CRISPR target sites not containing 1 or 2 mismatches.
(XLS)

Table S10 Three optimized candidate CRISPR target sequences were chosen for each exon of *Emx1* gene with a relatively low total number of off-target sites and POTs.
(XLS)

Table S11 The comparison of different CRISRP/Cas9 design tools.
(XLS)

File S1 **sgRNAcas9 software package.**
(RAR)

Text S1 **The mRNA sequence of human** ***Emx1*** **gene.**
(TXT)
